# Duffy Phenotype and *Plasmodium vivax* infections in Humans and Apes, Africa

**DOI:** 10.3201/eid1810.120120

**Published:** 2012-10

**Authors:** Richard Leighton Culleton, Pedro Eduardo Ferreira

**Affiliations:** Nagasaki University, Nagasaki, Japan (R.L. Culleton, P.E. Ferreira);; University of Algarve, Faro, Portugal (P.E. Ferreira);; and Karolinska Institutet, Stockholm, Sweden (P.E. Ferreira)

**Keywords:** Malaria, Africa, *Plasmodium vivax*, Duffy negativity, great apes, zoonosis, parasites, vector-borne infections

**To the Editor:** Benign tertian malaria, caused by *Plasmodium vivax,* has long been considered absent, or at least extremely rare, in western and central Africa. In these regions, 95%–99% of humans are of the Duffy negative phenotype, a condition that is thought to confer complete protection against the parasite during the blood stages of its life cycle (*1*,*2*). Sporadic reports throughout the latter half of the 20th century, however, have hinted at the presence of the parasite in these regions, the most convincing of which were the steady and consistent numbers of non-African travelers who returned to their countries of origin infected with malarial parasites that were subsequently identified as *P*. *vivax* (*2*).

More recently, evidence has emerged regarding the transmission of *P. vivax* in regions of Africa where the local human population is predominantly Duffy negative (*3*–*6*). In 4 (3.5%) of 155 patients from western Kenya (*6*), 7 (0.8%) of 898 persons from Angola (*4*), and 8 (8.2%) of 97 persons from Equatorial Guinea (*4*), *P. vivax* parasites were detected in the blood of apparently Duffy-negative persons, suggesting that the parasite might not be as absolutely dependent on the Duffy receptor for erythrocyte invasion as previously thought. These findings are supported by a report from Madagascar (where the human population is composed of a mixture of Duffy-positive and Duffy-negative persons), in which 42 (8.8%) of 476 Duffy-negative persons who had symptoms of malaria were reported to be positive for *P. vivax* by both microscopy and PCR (*7*). The prevalence of *P. vivax* in Duffy-negative persons was significantly lower than its prevalence in Duffy-positive persons residing in the same area, suggesting that Duffy negativity is a barrier to the parasite to some degree. Given the extremely high rates of malaria transmission in western and central Africa, a *P. vivax* parasite that could efficiently invade Duffy-negative erythrocytes would, presumably, become highly prevalent very rapidly.

With the exception of the cases reported from Angola and Kenya, which lie outside the area where the proportion of the population with Duffy negativity is highest, the reports of the transmission of *P. vivax* within predominantly Duffy-negative populations all come from regions inhabited by chimpanzees and gorillas (i.e., Democratic Republic of the Congo [*3*], Uganda [*4*], and Equatorial Guinea [*5*]). During our seroepidemiologic study from the Democratic Republic of the Congo, in which *P. vivax* sporozoite–specific antibodies were detected in ≈10% of the population, we found that women were significantly more likely than men to have been exposed to *P. vivax* sporozoites (*3*). Women in this region typically spend more time than men near the forest fringe, where they work in crop fields. This forest is within the known habitat range of the chimpanzee *Pan troglodytes* and the gorilla, *Gorilla gorilla gorilla*, both of which have been reported to be natural hosts of the malaria parasite *P. schwetzi*, which is a *P. vivax*–like or *P. ovale*–like parasite that might also be unable to invade the erythrocytes of persons who are Duffy negative (*8*). These animals have recently been shown to be infected occasionally with parasites that have mitochondrial genomes closely resembling those of *P. vivax* (*9*,*10*).

We have argued that, given the high malaria transmission rates in sub-Saharan Africa, it is plausible that the 1%–5% of the human population who are Duffy positive might maintain the transmission of the parasite (*2*). The discovery of *P. vivax* parasites (or *P. vivax*–like parasites) in the blood of African great apes leads to a question: could nonhuman primates in Africa be acting as Duffy-positive reservoirs of *P. vivax* in regions where the human population is almost entirely insusceptible? This possibility warrants further investigation. Given the increasing rarity of the great apes, however, their capacity to act as zoonotic reservoirs could be limited. It would be informative, in any case, to determine how the regions that *P. vivax*–positive travelers visit during their stay in Africa correspond with the ranges of chimpanzees and gorillas.

If African great apes do, indeed, constitute a zoonotic reservoir of *P. vivax* parasites, what are the repercussions for human health? Given that 95%–99% of humans possibly exposed to such a reservoir are Duffy negative, and therefore resistant to the parasite, these would appear to be slight. However, as humans encroach more frequently into ape habitats, the chances of humans encountering the parasite will increase. In the short term, the risks are probably limited to Duffy-positive persons who enter areas where apes are present, such as tourists and migrant workers.

## References

[1] Miller LH, Mason SJ, Clyde DF, McGinniss MH. The resistance factor to *Plasmodium vivax* in blacks. The Duffy-blood-group genotype, FyFy. N Engl J Med. 1976;295:302–4. 10.1056/NEJM197608052950602 778616

[2] Culleton RL, Mita T, Ndounga M, Unger H, Cravo P, Paganotti G, Failure to detect *Plasmodium vivax* in West and Central Africa by PCR species typing. Malar J. 2008;7:174. 10.1186/1475-2875-7-174 18783630 PMC2546428

[3] Culleton R, Ndounga M, Zeyrek FY, Coban C, Casimiro PN, Takeo S, Evidence for the transmission of *Plasmodium vivax* in the Republic of the Congo, West Central Africa. J Infect Dis. 2009;200:1465–9. 10.1086/644510 19803728

[4] Dhorda M, Nyehangane D, Renia L, Piola P, Guerin PJ, Snounou G. Transmission of *Plasmodium vivax* in south-western Uganda: report of three cases in pregnant women. PLoS ONE. 2011;6:e19801. 10.1371/journal.pone.0019801 21603649 PMC3094453

[5] Mendes C, Dias F, Figueiredo J, Mora VG, Cano J, de Sousa B, Duffy negative antigen is no longer a barrier to *Plasmodium vivax*–molecular evidences from the African West Coast (Angola and Equatorial Guinea). PLoS Negl Trop Dis. 2011;5:e1192. 10.1371/journal.pntd.0001192 21713024 PMC3119644

[6] Ryan JR, Stoute JA, Amon J, Dunton RF, Mtalib R, Koros J, Evidence for transmission of *Plasmodium vivax* among a Duffy antigen negative population in western Kenya. Am J Trop Med Hyg. 2006;75:575–81. 17038676

[7] Ménard D, Barnadas C, Bouchier C, Henry-Halldin C, Gray LR, Ratsimbasoa A, *Plasmodium vivax* clinical malaria is commonly observed in Duffy-negative Malagasy people. Proc Natl Acad Sci U S A. 2010;107:5967–71. 10.1073/pnas.0912496107 20231434 PMC2851935

[8] Coatney GR, Collins WE, Warren M, Contacos PG. *Plasmodium schwetzi*. In: Coatney GR, Collins WE, Warren M, Contacos PG, editors. The primate malarias. Bethesda (MD): US Department of Health, Education, and Welfare; 1971. p. 141–52.

[9] Krief S, Escalante AA, Pacheco MA, Mugisha L, Andre C, Halbwax M, On the diversity of malaria parasites in African apes and the origin of *Plasmodium falciparum* from Bonobos. PLoS Pathog. 2010;6:e1000765. 10.1371/journal.ppat.1000765 20169187 PMC2820532

[10] Liu W, Li Y, Learn GH, Rudicell RS, Robertson JD, Keele BF, Origin of the human malaria parasite *Plasmodium falciparum* in gorillas. Nature. 2010;467:420–5. 10.1038/nature09442 20864995 PMC2997044

